# Reflex of the Gracilis in Brain Death

**DOI:** 10.3390/diagnostics12102422

**Published:** 2022-10-07

**Authors:** Han Uk Ryu, Sang Yeon Kim, Hyun Goo Kang

**Affiliations:** 1 Department of Neurology, Jeonbuk National University Medical School and Hospital, Jeonju 54907, Korea; 2 Research Institute of Clinical Medicine of Jeonbuk National University, Biomedical Research Institute of Jeonbuk National University Hospital, Jeonju 54907, Korea

**Keywords:** brain death, reflex, movement

## Abstract

In patients with brain death, reflex movements originating from spinal reflexes are observed intermittently. Generally, they can occur under hypoxic stimuli such as when the ventilator is removed, under physical stimuli such as bending the neck, or under hypotension. Finger- and toe-jerk responses are commonly observed reflex movements that can occur in patients with brain death. In addition, the Lazarus sign, known as the most dramatic reflex movement, appears mainly in the upper extremities (e.g., the hands and arms) and in the distal lower extremities (e.g., the soles and toes). This case showed a reflex movement that was triggered by the contraction of the gracilis, a proximal muscle in the lower extremities, with only a gentle stimulus on the sole.

A 41-year-old man with a history of liver cirrhosis over 10 years, who had vomited and lost consciousness in a car in front of a factory, was transported to the emergency room. On admission, he was in a semicomatose state, and his Glasgow Coma Scale score was 4. Brain computed tomography performed within 10 minutes of his arrival showed intracerebral hemorrhage in his frontal lobe, intraventricular hemorrhage, and subarachnoid hemorrhage throughout his brain, including the basal cistern (Figure 1A). The Modified Fisher Score was 4 and the Hunt–Hess grade was 5. The patient fell into a coma on the ninth day of admission and lost his pupillary reflex, corneal reflex, oculo-cephalic reflex, vestibulo-ocular reflex, cilio-spinal reflex, gag reflex, and cough reflex. The intracerebral artery was not observed in brain magnetic resonance angiography (Figure 1B), and electroencephalography showed electrocerebral silence even at 2 µV/mm sensitivity (Figure 1C). During the neurological examination to determine brain death, the brain-stem reflex was not observed. However, when the left sole of his foot was stimulated, a reflex movement of the skin was subtended by a muscle contraction of the medial part of the left thigh (middle third and lower third). The visible contraction can be attributed to the gracilis muscle (Supplementary Video). No other spinal reflex was observed. 

In patients with brain death, reflex movements originating from spinal reflexes are observed intermittently. Generally, these movements can occur under hypoxic stimuli such as when the ventilator is removed, under physical stimuli such as bending the neck, or under hypotension [1]. Finger- and toe-jerk responses are commonly observed as reflex movements that can occur in patients with brain death [2]. In addition, the Lazarus sign, known as the most dramatic reflex movement, mainly appears in the upper extremities (e.g., the hands and arms) and in the distal lower extremities (e.g., the soles and toes) [2,3]. This patient showed a reflex movement that was triggered by the contraction of the left gracilis muscle, a proximal muscle in the lower extremities, with only a gentle stimulus on the left sole. Although the exact mechanism is unknown, a possible theory could be considered regarding the anatomy of nerve innervation. The medial plantar nerve that originates from the L4 and L5 spinal nerve roots and innervates to the sole may have contributed as an afferent pathway. The gracilis muscle, innervated by the obturator nerve, originates from the L2–L4 spinal nerve roots and may have contracted through an efferent pathway. An animal study showed that sustained hypoxia can lead to a significant increase in excitatory postsynaptic currents in tonically active motor neurons [4]. The hypothesized mechanism of extrasegmental spinal hyperexcitability is that hypoxia increased the synaptic strength and induced physiologic changes in tonically active gracilis motor neurons. 

The sudden presentation of an unusual muscle contraction can be observed in the case of brain death. It may be helpful to be aware of this gracilis muscle reflex when clinicians have to make rapid and accurate decisions regarding brain death, especially when the patient is a candidate for organ and tissue donation.

## Figures and Tables

**Figure 1:**
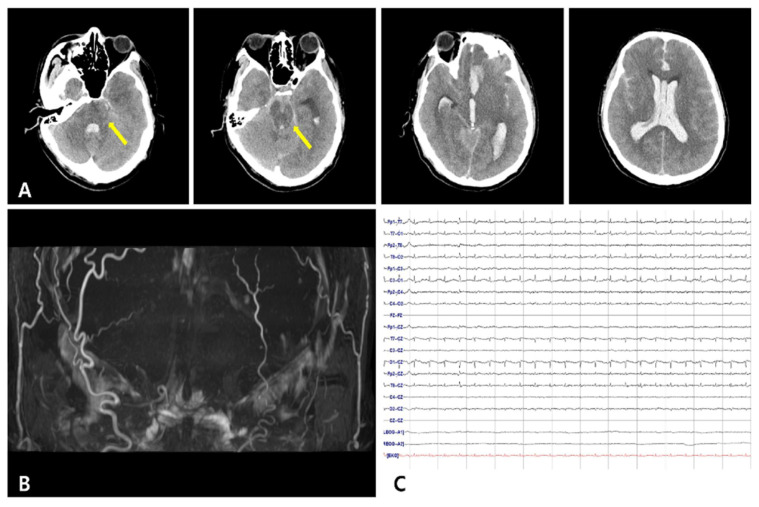
Radiologic findings and electroencephalography of the patient. (**A**) Brain computed tomography shows intracerebral hemorrhage in the left frontal lobe and massive intraventricular hemorrhage with subarachnoid hemorrhage including the basal cistern. Hypodense lesions in the brain stem were observed due to hypoxic brain damage (arrow). (**B**) Brain magnetic resonance angiography of the patient shows no cerebral blood flow. (**C**) There is no identifiable cerebral activity in any lead throughout the recording, even at 2 µV/mm sensitivity.

## Data Availability

Data available on request due to restrictions e.g., privacy or ethical.

## Supplementary Materials

The following supporting information can be downloaded at: https://www.mdpi.com/article/10.3390/diagnostics12102422/s1.
